# Functional Polymorphism in the *NFE2L2* Gene Associated With Tuberculosis Susceptibility

**DOI:** 10.3389/fimmu.2021.660384

**Published:** 2021-05-24

**Authors:** Guiyi Ji, Miaomiao Zhang, Qianqian Liu, Shouquan Wu, Yu Wang, Guo Chen, Andrew J. Sandford, Jian-Qing He

**Affiliations:** ^1^ Department of Respiratory and Critical Care Medicine, West China Hospital, Sichuan University, Chengdu, China; ^2^ Health Management Center, West China Hospital, Sichuan University, Chengdu, China; ^3^ Department of Respiratory Diseases, Chengdu Municipal First People’s Hospital, Chengdu, China; ^4^ Division of Geriatrics, Sichuan Provincial People’s Hospital, Chengdu, China; ^5^ Centre for Heart Lung Innovation, St. Paul’s Hospital, University of British Columbia, Vancouver, BC, Canada

**Keywords:** *NFE2L2*, polymorphism, tuberculosis, latent tuberculosis infection, susceptibility

## Abstract

**Background:**

Nuclear transcription factor erythroid 2 p45-related factor 2 (Nrf2), encoded by *NFE2L2*, functions as a key transcription factor and regulates expression of antioxidant genes. Our study aimed to investigate the association of single nucleotide polymorphisms of *NFE2L2* with tuberculosis (TB) and latent tuberculosis infection (LTBI) and the underlying causal mechanisms.

**Methods:**

1950 unrelated Chinese Han participants were included in our two independent study groups. Five tag polymorphisms were selected and genotyped. The functional effects of the rs13005431 polymorphism were confirmed by dual-luciferase reporter assays and mRNA level comparisons.

**Results:**

Rs13005431_C and rs2364723_G were associated with increased TB susceptibility (*P* = 0.010 and *P* = 0.041) after adjustment for confounding factors. rs6726395_A was associated with increased risk of active TB (*P*=0.035) in a comparison with the LTBI group. The frequency of haplotype rs1049751- rs13005431 AC was higher in the TB group (*P* =0.013), while frequency of haplotype AT was higher in the healthy control group (*P* =0.025). The luciferase activity of a plasmid with the rs13005431C-promoter was significantly lower than that of the rs13005431T-promoter. In addition, neutrophils with the CC/TC genotypes which were activated by GM-CSF showed a decreased level of *NFE2L2* mRNA when compared with the rs13005431 TT genotype.

**Conclusions:**

Our study suggests that allele C of rs13005431 might increase the susceptibility to TB by down-regulating the transcriptional activity of *NFE2L2*.

## Introduction

Tuberculosis (TB) is a disease caused by *Mycobacterium tuberculosis* (M.TB), which infects approximately one-third of the population worldwide, and remains one of the most important public health problems (1). In 2018, there were an estimated 10 million cases and 1.3 million deaths from tuberculosis worldwide (2). The aim of the End TB Strategy is to achieve a 95% reduction in TB deaths and a 90% reduction in the TB incidence rate by 2035 (2). Thus, great efforts are urgently needed to strengthen the prevention, diagnosis and treatment of TB.

As is well known, encountering M.TB leads to several possible outcomes including M. TB clearance, primary TB, latent TB infection (LTBI) and active TB. It has been suggested that 5-10% of LTBI individuals will progress to active TB during their lifetime (3). Management of LTBI will be required to implement the End TB Strategy (4). Screening and preventive treatment of LTBI have been recommended in populations with high risk (5). There are two available methods for LTBI diagnosis: tuberculin skin test (TST) and Interferon-gamma release assays (IGRAs) (including the Quantiferon GIT Assay and T-SPOT.TB). Although guidelines suggest that either the TST or IGRAs may be used to diagnose LTBI (5), IGRAs have been shown to have higher specificity and sensitivity (6) with no bacille Calmette–Guérin (BCG) vaccination interference and much less environmental mycobacteria interference than that of TST.

Previous studies have suggested that TB is associated with oxidative and antioxidant responses (7, 8). Nuclear factor-erythroid 2 (NF-E2)-related factor 2 (Nrf2), encoded by the *NFE2L2* gene, functions as a critical transcription factor in the anti-oxidation process and usually is repressed by Keap1. Nrf2 enters the nucleus upon activation by oxidative stress (9, 10), binds to the GCTGAGTCA site of the antioxidant response element in the promoter of antioxidant phase II genes and promotes the expression of these genes (11, 12). Several studies have revealed a relationship between Nrf2 and M.TB infection. It was reported that Nrf2 participated in autophagy (13), the antioxidant response in M. TB-infected guinea pigs (14), and the reduction of granulomas in Nrf2-deficient mice when infected with M.TB (15).

Recently, oxidant/antioxidant related genes such as *FMO2* and *CYBA* have been associated with TB susceptibility (16, 17). However, there have been no reports of an association between *NFE2L2* polymorphisms and risk of LTBI and TB. Therefore, we carried out a discovery study and a replication study to determine the relationship of *NFE2L2* variants with susceptibility to TB and LTBI in the Chinese Han population. We also performed dual-luciferase reporter assays and compared *NFE2L2* mRNA levels of neutrophils with different genotypes to validate the association findings.

## Materials and Methods

### Study Population

In total, 1950 unrelated Chinese Han participants, consisting of 636 TB patients and 608 healthy controls in the discovery study as well as 301 TB patients, 201 LTBI subjects and 204 uninfected healthy controls (HC) in the replication study, were consecutively recruited from the West China Hospital between July 2013 and August 2017. For the discovery study, the diagnosis of TB was based upon the following criteria: histopathological evidence of TB disease and/or culture positivity for MTB and/or smear positivity for MTB in at least two separate specimens and/or radiological and clinical findings consistent with TB, with positive clinical response to anti-TB therapy (18). TB cases were divided into two subgroups: (1) pulmonary TB patients (PTB, pathological changes limited to the lung) and (2) extra-pulmonary patients (EPTB, pathological changes involving other tissues or organs merely or in combination with the lungs). The healthy controls who did not have a history or evidence of TB on the basis of their syndromes and radiographic examination results were enrolled during their routine health examination in the West China Hospital. For the replication study, both the uninfected HC group and LTBI participants were close contacts of PTB patients. LTBI was defined as adults (age > 18) with a documented positive result of IGRAs and negative results of radiological and clinical manifestations. HC individuals were defined as adults with negative results of IGRAs, radiological and clinical findings. The inclusion criteria of TB patients enrolled in the replication study were the same as the discovery study. Another 60 healthy volunteers were recruited for comparison of mRNA levels of neutrophils. All participants diagnosed with HIV infection, diabetes mellitus, autoimmune disorders, tumors or treated with immunosuppressive drugs were excluded.

### Selection/Genotyping of *NFE2L2* Gene SNPs

*NFE2L2* is located on chromosome 2 (2q31) and has five exons and four introns. TagSNPs were selected from the region 3,000 base pairs upstream to 300 base pairs downstream of the *NFE2L2* gene based on the Chinese Han Beijing data of the HapMap database (http://hapmap.ncbi.nlm.nih.gov, HapMap Data Rel 27 Phase II + III, Feb09, on NCBI B36 assembly, dbSNP b126) by Haploview software 4.2. Using criteria of minor allele frequency (MAF) ≥ 5% and the Tagger pairwise method (r² ≥0.8), five tagSNPs of *NFE2L2* (rs10497511, rs2364723, rs13005431, rs6726395 and rs1962142), representing eleven SNPs with MAF ≥ 0.05 in the covered gene region, were selected for genotyping.

A total of 5 ml peripheral venous blood was collected from each subject and genomic DNA was extracted using the AxyPrep genomic DNA Mini kits (Axygen, USA) following the manufacturer’s protocol. SNPs were genotyped on the MassArray Analyzer 4 system (Sequenom,USA). All probes and primers are available upon request. 5% samples were genotyped twice for quality control. The genotyping related primers were listed in **Supplementary Table 1**.

### Plasmid Constructs

Polymerase chain reaction (PCR) was performed to amplify a 651bp sequence surrounding rs13005431 from genotyped genomic DNA with the TT genotype of rs13005431. Primers were designed using Primer-BLAST (www.ncbi.nlm.nih.gov/tools/primer-blast/) and sequences for restriction sites *Nhe*I and *Xho*I were introduced (**Supplementary Table 2**). Both gel purified PCR product and pGL3-promoter reporter vector (Promega, Madison, WI, USA) were digested by *Nhe*I and *Xho*I restriction enzymes (Takara Bio Inc., Kusatsu, Japan) at 37°C for 4 hours and then ligated by T4 DNA ligase (TaKara Bio Inc.) at 16°C for 8 hours. After transforming into competent *E. coli* DH5α cells and extraction by plasmid miniprep kit (TianGen, Beijing, China), the recombinant plasmid vector was verified by direct sequencing and named PGL3-rs13005431T-promoter (**Supplementary Figure 1**). A site-directed mutagenesis strategy was applied to acquire another recombinant plasmid vector of PGL3-rs13005431C-promoter (see **Supplementary Table 2** for primer sequences) which was confirmed by DNA sequencing.

### Cell Culture and Luciferase Assays

HEK293T cells were plated into 96-well culture plates at a density of 3.2×10^4^ cells/well and cultured for 48 hours to acquire 90% confluence at the time of transfection. The PGL3-promoter, PGL3-rs13005431T-promoter and PGL3-rs13005431C-promoter were transfected into the HEK293T cells together with PRL-CMV plasmid vectors which acted as an internal reference using Lipofectamine^®^ 2000 Reagent (Invitrogen (Thermo Fisher Scientific), Waltham, MA USA). Dual-luciferase reporter assays (Promega, USA) were carried out after 30h transfection on a GloMax™ 96 microplate luminometer. The transfection experiment was performed in triplicate. Results are presented as relative luciferase activity by dividing the firefly luciferase activity of each well by the *Renilla* luciferase activity. The normalized luciferase activity of recombinant plasmids was further normalized to the PGL3-promoter group.

### mRNA Levels of *NFE2L2* in Individuals With Different Genotypes of rs13005431

10 ml of peripheral venous blood in a heparin anticoagulant tube was obtained from 60 healthy volunteers. Neutrophils were isolated using Ficoll gradient density centrifugation and confirmed by Wright staining and flow cytometry (CD11b, CD16). The cells were then cultured in 24-well culture plates at a density of 2×10^6^ cells/well for 3 hours, activated or not by GM-CSF (in a final concentration of 1 ng/ml). The Trizol method was then used to extract the total RNA of the neutrophils. Reverse transcription was conducted using the PrimeScript™ RT Reagent Kit with gDNA Eraser (Takara Bio Inc.). Real-time PCR for *NFE2L2* was then performed on an ABI7300 Sequence Detection System (Applied Biosystems (Thermo Fisher Scientific), Waltham, MA USA) using SYBR Premix Ex Taq II (Takara Bio Inc.), with *B2M* as the house-keeping gene (19). Primers for real-time PCR are shown in the **Supplementary Table 2**.

### Statistical Analyses

Experimental data were analyzed using the Statistical Package for Social Sciences version 17.0 (SPSS, Chicago, IL, USA). Distributions of clinical characteristics between the control group and TB patients were evaluated by the chi-squared test and Fisher exact probability for categorical variables and the student’s t-test for continuous variables. *P* values, Odds Ratios (OR) and 95% confidence intervals (95%CI) of association between SNPs and TB susceptibility were calculated by binary or multinomial logistic regression under four genetic models (allelic, additive, dominant and recessive). Linkage disequilibrium (LD) analysis and haplotype blocks were created by Haploview software 4.2 (20), while p values, ORs, 95%CIs and the global test of haplotype analysis were calculated using the SHEsis program (21). Genotype and allele frequencies of SNPs in the control group were assessed to determine whether they conformed to Hardy-Weinberg equilibrium (HWE). Gene-environment interaction was detected using the multifactor dimensionality reduction (MDR) constructive induction algorithm. SNP-by-sex additive interactions by the method of Andersson and multiplicative interactions by logistic regression in the discovery study (22). Normalized luciferase activity and relative mRNA levels were evaluated by the student’s t-test. A statistically significant difference was indicated by a two-sided P-value < 0.05.

## Results

### Demographic and Clinical Characteristics

**Table 1** shows the characteristics of all the study subjects. In the discovery study, there was no significant difference in the distribution of gender and age between the case and control groups. However, smoking status was statistically different (*P* = 0.003). In the replication study, although gender distribution was similar among the three groups, age significantly differed between groups. Smoking status and TB types of the participants in the replication study were not available.

**Table 1:** Characteristics of the discovery and replication studies.

Study	Characteristics	Control (n = 608) n (%)		TB (n = 636) n (%)	p value	
Discovery study	Gender				0.654	
	male	302 (49.7)		324 (50.9)	–	
	female	306 (50.3)		312 (49.1)	–	
	Age (mean ± SD)	37.1 ± 15.7		36.8 ± 15.7	0.677	
	Smoking status				**0.003**	
	Current or ever smoking	141 (23.2)		195 (30.7)	–	
	non-smoking	467 (76.8)		441 (69.3)	–	
	TB type					
	PTB	–		276 (43.4)	–	
	EPTB	–		360 (56.6)	–	
Replication study		Uninfected HC (n = 204) n(%)	LTBI (n = 201) n(%)	TB (n = 301) n(%)	Uninfected HC vs. LTBI	LTBI vs. TB
	Gender				0.735	0.282
	male	93 (46.6)	95 (47.3)	157 (52.2)	–	–
	female	111 (54.4)	106 (52.7)	144 (47.8)	–	–
	Age (mean ± SD)	45.7 ± 14.9	49.1 ± 15.9	39.1 ± 16.8	**0.027**	**<0.001**

TB, tuberculosis; SD, standard deviation; PTB, pulmonary tuberculosis, pathological changes limited to lung; EPTB, extra-pulmonary tuberculosis, pathological changes involving other tissues or organs merely or in combination with the lungs; HC, healthy controls; LTBI, latent tuberculosis infection. Bold values: P-value < 0.05.

**Figure 1:**
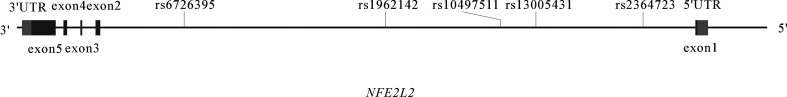
*NFE2L2* gene structure and location of five tagSNPs (rs10497511, rs2364723, rs13005431, rs6726395 and rs1962142).

The locations of the five tagSNPs genotyped are presented in **Figure 1**. The genotype call rates varied from 99.7% to 100% and the genotype reproducibility was 100%. All SNPs in the control group of both studies were in HWE.

### Association Between the Five tagSNPs of *NFE2L2* and Susceptibility to TB and LTBI in the Discovery and Replication Study

In the discovery study, we analyzed the data between TB patients and healthy controls. As shown in **Table 2**, allele C of rs13005431 and allele G of rs2364723 were associated with increased TB risk (*P* = 0.010 and *P* = 0.041). In stratified analyses, the deleterious effect of rs13005431 C was still seen in the non-smoker, EPTB and female subgroups (*P* = 0.003; *P* = 0.015 and *P*<0.001, respectively). In addition, rs2364723 G still demonstrated a relationship with increased susceptibility to TB in the non-smoker and female subgroups (*P* = 0.022 and *P* = 0.004). In addition, allele A of rs6726395 was demonstrated to be a risk factor associated with TB risk in the female subgroup (*P* = 0.003). There was no significant association observed between the other two tagSNPs (rs10497511 and rs1962142) and TB susceptibility (**Table 2**).

**Table 2:** Significant associations between five tagSNPs (rs13005431, rs2364723, rs6726395, rs10497511 and rs1962142) and tuberculosis risk in the discovery study and associations stratified by gender, smoking status and TB types.

SNPs	Genetic models	Genotypes/Alleles	Controls, n (%)	TB, n (%)	*P* ^a^ value	OR (95% CI)^a^	Female subgroup		Nonsmoking subgroup		EPTB subgroup	
							*P* ^a^ value	OR (95% CI)^a^	*P* ^a^ value	OR (95% CI)^a^	*P* ^a^ value	OR (95% CI)^a^
rs13005431	allelic	T	1060 (87.3)	1061 (83.7)	-	Reference	-	Reference	-	Reference	-	Reference
		C	154 (12.7)	207 (16.3)	**0.010**	1.35 (1.07-1.69)	**<0.001**	1.91 (1.37-2.66)	**0.003**	1.49 (1.15-1.95)	**0.015**	1.38 (1.07-1.79)
	additive	TT	461 (75.9)	447 (70.5)	**-**	Reference	**-**	Reference	**-**	Reference	**-**	Reference
		TC	138 (22.7)	167 (26.3)	**0.026**	2.58 (1.12-5.93)	**0.024**	4.44 (1.22-16.14)	0.098	2.20 (0.87-5.58)	**0.019**	2.92 (1.19-7.17)
		CC	8 (1.3)	20 (3.2)	0.090	1.26 (0.97-1.63)	**0.002**	1.83 (1.25-2.68)	**0.011**	1.49 (1.10-2.03)	0.148	1.25 (0.92-1.70)
	dominant	TT/TC+CC			**0.029**	1.33 (1.03-1.71)	**<0.001**	1.96 (1.35-2.84)	**0.005**	1.54 (1.14-2.08)	**0.049**	1.35 (1.00-1.81)
	recessive	TT+TC/CC			**0.036**	2.44 (1.06-5.59)	**0.041**	3.82 (1.05-13.83)	0.146	1.99 (0.79-5.04)	**0.027**	2.75 (1.13-6.74)
rs2364723	allelic	C	633 (52.1)	605 (47.6)	**-**	Reference	**-**	Reference	**-**	Reference	**-**	Reference
		G	583 (47.9)	665 (52.4)	**0.041**	1.18 (1.01-1.38)	**0.004**	1.39 (1.11-1.74)	**0.022**	1.24 (1.03-1.49)	0.100	1.17 (0.97-1.41)
	additive	CC	166 (27.3)	148 (23.3)	**-**	Reference	**-**	Reference	**-**	Reference	**-**	Reference
		CG	301 (49.5)	309 (48.7)	**0.043**	1.38 (1.01-1.90)	**0.005**	1.92 (1.22-3.03)	**0.022**	1.54 (1.07-2.24)	0.100	1.37 (0.94-1.99)
		GG	141 (23.2)	178 (28.0)	0.295	1.16 (0.88-1.52)	0.050	1.48 (1.00-2.17)	0.125	1.28 (0.93-1.76)	0.183	1.25 (0.90-1.73)
	dominant	CC/CG+GG			0.115	1.23 (0.95-1.59)	**0.011**	4.29 (1.21-15.27)	**0.043**	1.36 (1.01-1.84)	0.108	1.29 (0.95-1.75)
	recessive	CC+CG/GG			0.083	1.26 (0.97-1.63)	**0.037**	1.49 (1.02-2.16)	0.084	1.31 (0.97-1.78)	0.282	1.18 (0.87-1.60)
rs6726395	allelic	G	743 (61.1)	742 (58.3)	**-**	Reference	**-**	Reference	**-**	Reference	**-**	Reference
		A	473 (38.9)	530 (41.7)	0.231	1.10 (0.94-1.30)	**0.003**	1.42 (1.12-1.78)	0.173	1.14 (0.94-1.38)	0.221	1.13 (0.93-1.36)
	additive	GG	226 (37.2)	219 (34.4)	**-**	Reference	**-**	Reference	**-**	Reference	**-**	Reference
		AG	291 (47.9)	304 (47.8)	0.211	1.24 (0.89-1.73)	**0.008**	1.93 (1.19-3.12)	0.205	1.29 (0.87-1.92)	0.194	1.29 (0.88-1.91)
		AA	91 (15.0)	113 (17.8)	0.621	1.06 (0.83-1.36)	**0.022**	1.51 (1.06-2.14)	0.335	1.15 (0.87-1.53)	0.649	1.07 (0.80-1.43)
	dominant	GG/AG+AA			0.397	1.11 (0.88-1.40)	**0.005**	1.60 (1.15-2.23)	0.219	1.18 (0.9-1.55)	0.406	1.12 (0.85-1.48)
	recessive	GG+AG/AA			0.250	1.20 (0.88-1.62)	0.058	1.53 (0.99-2.37)	0.337	1.19 (0.83-1.72)	0.221	1.24 (0.88-1.77)
rs10497511	allelic	A	931 (76.7)	959 (75.6)	–	Reference	–	Reference	–	Reference	–	Reference
		G	283 (23.3)	309 (24.4)	0.699	1.04 (0.86-1.25)	0.225	1.18 (0.90-1.55)	0.595	1.06 (0.85-1.32)	0.483	1.08 (0.87-1.34)
	additive	AA	353 (58.2)	361 (56.9)	–	Reference	–	Reference	–	Reference	–	Reference
		AG	225 (37.1)	237 (37.4)	0.584	1.16 (0.69-1.93)	0.383	1.44 (0.64-3.23)	0.590	1.19 (0.63-2.27)	0.743	1.11 (0.60-2.05)
		GG	29 (4.8)	36 (5.7)	0.934	1.01 (0.80-1.28)	0.306	1.19 (0.85-1.67)	0.754	1.05 (0.79-1.38)	0.459	1.11 (0.84-1.46)
	dominant	AA/AG+GG			0.820	1.03 (0.82-1.29)	0.242	1.21 (0.88-1.68)	0.666	1.06 (0.81-1.38)	0.443	1.11 (0.85-1.44)
	recessive	AA+AG/GG			0.588	1.15 (0.69-1.91)	0.474	1.34 (0.60-2.99)	0.620	1.17 (0.62-2.22)	0.842	1.06 (0.58-1.95)
rs1962142	allelic	G	953 (78.4)	992 (78.0)	–	Reference	–	Reference	–	Reference	–	Reference
		A	263 (21.6)	280 (22.0)	0.943	0.99 (0.82-1.20)	0.585	1.08 (0.82-1.42)	0.892	1.02 (0.81-1.28)	0.503	1.08 (0.87-1.35)
	additive	GG	369 (60.7)	389 (61.2)	–	Reference	–	Reference	–	Reference	–	Reference
		AG	215 (35.4)	214 (33.6)	0.499	1.21 (0.70-2.09)	0.987	0.99 (0.45-2.21)	0.436	1.32 (0.66-2.65)	0.331	1.36 (0.73-2.55)
		AA	24 (3.9)	33 (5.2)	0.507	0.92 (0.73-1.17)	0.439	1.14 (0.81-1.61)	0.687	0.94 (0.71-1.25)	0.883	1.02 (0.77-1.35)
	dominant	GG/AG+AA			0.671	0.95 (0.76-1.20)	0.478	1.13 (0.81-1.57)	0.863	0.98 (0.75-1.28)	0.691	1.06 (0.81-1.38)
	recessive	GG+AG/AA			0.428	1.25 (0.72-2.14)	0.888	0.95 (0.43-2.08)	0.399	1.35 (0.67-2.69)	0.336	1.35 (0.73-2.51)

TB, tuberculosis; OR, odds ratio; CI, confidence interval; EPTB, extra-pulmonary tuberculosis, pathological changes involving other tissues or organs merely or in combination with the lungs; allelic, allelic model; additive, additive model; dominant, dominant model; recessive, recessive model.

a Adjusting for cofounders including age, gender and smoking status in a binary logistic regression analysis model. Bold values: P-value < 0.05.

In the replication study, we analyzed the data among TB patients, LTBI subjects and uninfected healthy controls using a multinomial logistic regression analysis model. When comparing the LTBI group with TB patients, only allele A of rs6726395 was associated with increased TB risk (*P* = 0.035) (**Table 3**). When the HC group was compared with the LTBI group, none of the tagSNPs was associated with susceptibility to TB infection. In addition, we combined LTBI subjects and uninfected healthy controls as a healthy control group. When comparing the healthy control group with TB patients, we still seen that rs6726395 A was associated with increased TB risk (*P* = 0.028) (**Table 4**).

**Table 3:** Associations between five tagSNPs (rs13005431, rs2364723, rs6726395, rs10497511 and rs1962142) and risk of LTBI and TB in the replication study.

SNPs	Genetic models	Genotypes/	Controls, n (%)		TB, n (%)	uninfected HC vs. LTBI			LTBI vs. TB	
		alleles	uninfected HC, n (%)	LTBI, n (%)		*P* ^f^	*P* ^a^	OR (95% CI)^a^	*P* ^a^	OR (95% CI)^a^
rs13005431	allelic	T	351 (86.0)	352 (88.0)	505 (83.9)		**-**	Reference	**-**	Reference
		C	57 (14.0)	48 (12.0)	97 (16.1)		0.387	0.83 (0.55-1.26)	0.077	1.42 (0.96-2.08)
	additive	TT	150 (73.5)	155 (77.5)	212 (70.4)		**-**	Reference	**-**	Reference
		TC	51 (25.0)	42 (21.0)	81 (27.0)		0.966	0.97 (0.19-4.89)	0.377	1.87 (0.47-7.52)
		CC	3 (1.5)	3 (1.5)	8 (2.7)	0.473	0.320	0.79 (0.49-1.26)	0.114	1.43 (0.92-2.22)
	dominant	TC+CC/TT					0.335	0.80 (0.51-1.26)	0.084	1.46 (0.95-2.24)
	recessive	CC/TT+TC				0.652	0.981	1.02 (0.20-5.15)	0.444	1.72 (0.43-6.87)
rs2364723	allelic	C	202 (49.5)	199 (49.8)	284 (47.2)		**-**	Reference	**-**	Reference
		G	206 (50.5)	201 (50.3)	318 (52.8)		0.842	0.97 (0.74-1.28)	0.223	1.18 (0.91-1.53)
	additive	CC	51 (25.0)	54 (27.0)	70 (23.3)		**-**	Reference	**-**	Reference
		CG	100 (49.0)	91 (45.5)	144 (47.8)		0.837	0.95 (0.55-1.62)	0.225	1.37 (0.82-1.28)
		GG	53 (26.0)	55 (27.5)	87 (29.0)		0.456	0.83 (0.52-1.35)	0.237	1.32 (0.83-2.08)
	dominant	CG+CC/GG					0.549	0.87 (0.56-1.36)	0.182	1.34 (0.87-2.05)
	recessive	CC/GG+CG					0.785	1.06 (0.68-1.66)	0.524	1.14 (0.78-1.73)
rs6726395	allelic	G	255 (62.5)	255 (63.8)	350 (58.1)		**-**	Reference	**-**	Reference
		A	153 (37.5)	145 (36.3)	252 (41.9)		0.609	0.93 (0.70-1.24)	**0.035**	1.34 (1.02-1.75)
	additive	GG	80 (39.2)	83 (41.5)	109 (36.2)		**-**	Reference	**-**	Reference
		AG	95 (46.6)	89 (44.5)	132 (43.9)		0.721	0.90 (0.49-1.64)	**0.040**	1.79 (1.03-3.11)
		AA	29 (14.2)	28 (14.0)	60 (20.0)		0.553	0.88 (0.58-1.35)	0.326	1.23 (0.82-1.84)
	dominant	AG+AA/GG					0.542	0.88 (0.59-1.32)	0.114	1.36 (0.93-1.99)
	recessive	AA/GG+AG					0.885	0.96 (0.55-1.68)	0.069	1.60 (0.96-2.66)
rs10497511	allelic	A	307 (75.2)	297 (74.3)	446 (74.1)		**-**	Reference	**-**	Reference
		G	101 (24.8)	103 (25.8)	156 (25.9)		0.822	1.04 (0.75-1.43)	0.642	1.07 (0.80-1.45)
	additive	AA	116 (56.9)	110 (55.0)	166 (55.1)		**-**	Reference	**-**	Reference
		AG	75 (36.8)	77 (38.5)	114 (37.9)		0.969	1.02 (0.45-2.30)	0.574	1.24 (0.58-2.65)
		GG	13 (6.4)	13 (6.5)	21 (7.0)		0.752	1.07 (0.71-1.62)	0.884	1.03 (0.70-1.52)
	dominant	AA/AG+GG					0.769	1.06 (0.72-1.57)	0.763	1.06 (0.73-1.54)
	recessive	AA+AG/GG					0.979	0.99 (0.45-2.20)	0.587	1.23 (0.59-2.57)
rs1962142	allelic	G	324 (79.4)	309 (77.3)	470 (78.1)		**-**	Reference	**-**	Reference
		A	84 (20.6)	91 (22.8)	132 (21.9)		0.502	1.12 (0.80-1.57)	0.977	1.00 (0.73-1.36)
	additive	GG	129 (63.2)	118 (59.0)	183 (60.8)		**-**	Reference	–	Reference
		AG	66 (32.4)	73 (36.5)	104 (34.6)		0.954	1.03 (0.39-2.69)	0.696	1.20 (0.49-2.95)
		AA	9 (4.4)	9 (4.5)	14 (4.7)		0.369	1.21 (0.80-1.84)	0.711	0.93 (0.63-1.37)
	dominant	GG/AG+AA					0.401	1.19 (0.80-1.78)	0.816	0.96 (0.66-1.40)
	recessive	GG+AG/AA					0.933	0.96 (0.37-2.48)	0.648	1.23 (0.51-2.99)

Uninfected HC, uninfected healthy control; LTBI, latent tuberculosis infection; TB, tuberculosis; OR, odds ratio; CI, confidence interval; allelic, allelic model; additive, additive model; dominant, dominant model; recessive, recessive model.

^a^Adjusting for age and gender in a multinomial logistic regression analysis model.

^f^Fisher exact probability method for RXC contingency table. Bold values: P-value < 0.05.

**Table 4:** Significant associations between five tagSNPs (rs13005431, rs2364723, rs6726395, rs10497511 and rs1962142) and tuberculosis risk in the replication study and the whole study including discovery study and replication study.

SNPs	Genetic models	Genotypes/Alleles	Replication study				Discovery and replication study			
			Controls, n (%)	TB, n (%)	P^a^ value	OR (95% CI)^a^	Controls, n (%)	TB, n (%)	P^a^ value	OR (95% CI)^a^
rs13005431	allelic	T	703 (87.0)	505 (83.9)	**-**	Reference	1763 (87.2)	1566 (83.7)	**-**	Reference
		C	105 (13.0)	97 (16.1)	0.115	1.28 (0.94-1.75)	259 (12.8)	304 (16.3)	**0.002**	1.34 (1.12-1.60)
	additive	TT	305 (75.5)	212 (70.4)	**-**	Reference	766 (75.8)	659 (70.5)	**-**	Reference
		TC	93 (23.0)	81 (27.0)	0.284	1.84 (0.60-5.59)	231 (22.8)	248 (26.5)	**0.011**	2.33 (1.21-4.49)
		CC	6 (1.5)	8 (2.7)	0.210	1.26 (0.88-1.80)	14 (1.4)	28 (3.0)	**0.027**	1.27 (1.03-1.56)
	dominant	TT/TC+CC			0.147	1.30 (0.91-1.84)			**0.006**	1.33 (1.09-1.63)
	recessive	TT+TC/CC			0.330	1.74 (0.57-5.26)			**0.018**	2.20 (1.15-4.22)
rs2364723	allelic	C	401 (49.6)	284 (47.2)	**-**	Reference	1034 (92.0)	889 (47.5)	**-**	Reference
		G	407 (50.4)	318 (52.8)	0.183	1.16 (0.93-1.45)	990 (8.0)	983 (52.5)	**0.013**	1.18 (1.04-1.33)
	additive	CC	105 (26.0)	70 (23.3)	**-**	Reference	271 (26.8)	218 (23.3)	**-**	Reference
		CG	191 (47.3)	144 (47.8)	0.192	1.33 (0.87-2.04)	492 (48.6)	453 (48.4)	**0.014**	1.37 (1.07-1.76)
		GG	108 (26.7)	87 (29.0)	0.356	1.20 (0.82-1.76)	249 (24.6)	265 (28.3)	0.134	1.18 (0.95-1.48)
	dominant	CC/CG+GG			0.232	1.25 (0.87-1.79)			**0.038**	1.25 (1.01-1.53)
	recessive	CC+CG/GG			0.345	1.18 (0.84-1.67)			0.051	1.23 (1.00-1.50)
rs6726395	allelic	G	510 (63.1)	350 (58.1)	**-**	Reference	1253 (94.2)	1092 (58.3)	**-**	Reference
		A	298 (36.9)	252 (41.9)	**0.028**	1.29 (1.03-1.61)	771 (5.8)	782 (41.7)	**0.014**	1.18 (1.03-1.34)
	additive	GG	163 (40.3)	109 (36.2)	**-**	Reference	389 (38.4)	328 (35.0)	**-**	Reference
		AG	184 (45.5)	132 (43.9)	**0.024**	1.69 (1.07-2.66)	475 (46.9)	436 (46.5)	**0.013**	1.40 (1.08-1.83)
		AA	57 (14.2)	60 (20.0)	0.433	1.15 (0.82-1.62)	148 (14.7)	173 (18.5)	0.276	1.12 (0.92-1.36)
	dominant	GG/AG+AA			0.137	1.27 (0.93-1.75)			0.074	1.19 (0.98-1.43)
	recessive	GG+AG/AA			**0.034**	1.57 (1.04-2.37)			**0.025**	1.32 (1.04-1.68)
rs10497511	allelic	A	604 (74.8)	446 (74.1)	**-**	Reference	1535 (75.9)	1405 (75.1)	**-**	Reference
		G	204 (25.2)	156 (25.9)	0.482	1.09 (0.85-1.41)	487 (24.1)	465 (24.9)	0.485	1.05 (0.91-1.22)
	additive	AA	226 (55.9)	166 (55.1)	**-**	Reference	579 (57.3)	527 (56.4)	**-**	Reference
		AG	152 (37.6)	114 (37.9)	0.482	1.25 (0.67-2.35)	377 (37.3)	351 (37.5)	0.473	1.15 (0.78-1.71)
		GG	26 (6.4)	21 (7.0)	0.702	1.07 (0.77-1.48)	55 (5.4)	57 (6.1)	0.706	1.04 (0.86-1.25)
	dominant	AA/AG+GG			0.580	1.09 (0.80-1.49)			0.584	1.05 (0.88-1.26)
	recessive	AA+AG/GG			0.525	1.22 (0.66-2.26)			0.511	1.14 (0.78-1.67)
rs1962142	allelic	G	633 (78.3)	470 (78.1)	**-**	Reference	1586 (78.4)	1462 (78.0)	**-**	Reference
		A	175 (21.7)	132 (21.9)	0.688	1.06 (0.81-1.38)	438 (21.6)	412 (22.0)	0.758	1.02 (0.88-1.19)
	additive	GG	247 (61.1)	183 (60.8)	**-**	Reference	616 (60.9)	572 (61.0)	**-**	Reference
		AG	139 (34.4)	104 (34.6)	0.611	1.22 (0.57-2.58)	354 (35.0)	318 (33.9)	0.375	1.22 (0.79-1.88)
		AA	18 (4.5)	14 (4.7)	0.883	1.03 (0.74-1.42)	42 (4.2)	47 (5.0)	0.758	0.97 (0.80-1.18)
	dominant	GG/AG+AA			0.785	1.05 (0.76-1.44)			0.971	1.00 (0.83-1.20)
	recessive	GG+AG/AA			0.623	1.20 (0.57-2.53)			0.343	1.23 (0.80-1.89)

TB, tuberculosis; OR, odds ratio; CI, confidence interval; allelic, allelic model; additive, additive model; dominant, dominant model; recessive, recessive model.

^a^Adjusting for cofounders including age and gender in a binary logistic regression analysis model. Bold values: P-value < 0.05.

Furthermore, we combined the data of the two studies including the discovery study and the replication study. As shown in **Table 4**, allele C of rs13005431, allele G of rs2364723, and allele A of rs6726395 were associated with increased TB risk (*P* = 0.002; *P* = 0.013 and *P* = 0.014, respectively). There was no significant association observed between the other two tagSNPs (rs10497511 and rs1962142) and TB susceptibility.

### LD and Haplotype Analysis in the Discovery and Replication Study

The LD analysis (**Supplementary Figure 2**) demonstrated mild-to-moderate levels of LD between the five tagSNPs of NFE2L2. For the discovery study, haplotype analyses of the two blocks are shown in **Supplementary Table 3**. The frequency of haplotype rs1049751-rs13005431 AC was significantly higher in the TB group (*P* = 0.013), while the frequency of haplotype AT was higher in the control group (*P* = 0.025) and there was a significant global result (*P* = 0.025). For the replication study, no significant haplotype effect was observed.

### Association Between Gene-Environment/SNP-by-Sex Interactions and TB Susceptibility

The results of gene-environment interactions after MDR analysis are shown in **Supplementary Table 4**. In the discovery study, smoking-rs13005431 formed the best interaction model with 55.69% testing balanced accuracy and 10/10 cross-validation consistency. Smoking-sex-rs13005431 formed an interaction model with 55.03% testing balanced accuracy and 8/10 cross-validation consistency. After 1000-fold permutation testing, both models were found to be significant (*P* = 0.001-0.002 and *P* = 0.023, respectively). As shown in **Supplementary Figure 3**, smoking status formed the high-risk models regardless the genotypes of rs13005431, non-smoking status and rs13005431 TT formed the low-risk model, while non-smoking status and rs13005431 CC/TC formed the high-risk models. As shown in **Supplementary Table 5**, the only positive additive interaction was observed between female sex and rs13005431 TC+CC/C genotypes. In addition, there were suggestive positive multiplicative interactions of three SNPs (rs13005431, rs2364723 and rs6726395) with female sex under two genetic models.

### Bioinformatics Prediction

We predicted the potential functional significance of SNPs from three aspects including protein coding, splicing regulation and transcription regulation (**Supplementary Table 6**). The influence factors of transcription regulation can be judged by three terms as following: 1) prediction of transcription factor binding sites altering using TRANSFAC or JASPAR database; 2) other factors affecting transcription regulation, such as high sensitive site of DNase I, histone modification, according to the comprehensive evaluation by GLODEN path; 3) judgment of evolutionary conservatism by PhastCons. According to the predicted results, rs13005431 was identified as a candidate SNP for further functional identification.

### Effect of Allele T/C of rs13005431 on Promoter Activity

**Figure 2:**
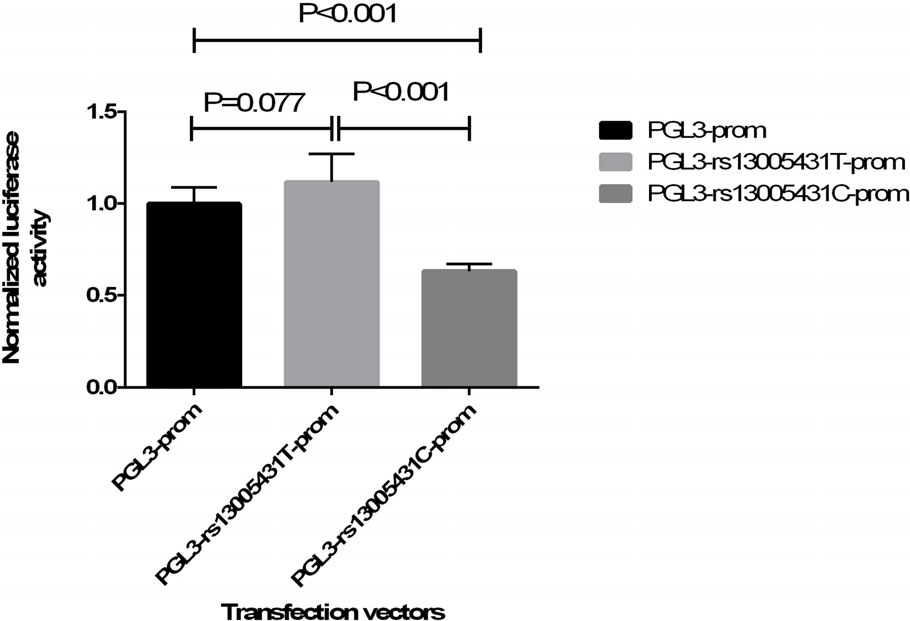
Normalized luciferase activity of three transfection vectors. The relative luciferase activity is presented as the firefly luciferase activity of each well divided by the *Renilla* luciferase activity. The normalized luciferase activity was expressed as a ratio to the mean relative luciferase activity of the PGL3-promoter group by each relative luciferase activity.

rs13005431, located in the first intron of *NFE2L2*, is outside of the promoter region. However, this SNP might disrupt an enhancer or silencer sequence and thus influence promoter activity indirectly. As previously reported, variants in the first intron might influence gene activity (23). Moreover, rs13005431 was reported to be a cis-eQTL (expression quantitative trait locus) of *NFE2L2* in whole blood (*P* = 1.978×10^-4^) (24). Therefore, the dual-luciferase reporter assay was used to investigate the regulatory role of rs13005431. As shown in **Figure 2**, the normalized luciferase activity of PGL3-rs13005431C-promoter was significantly lower than the PGL3-rs13005431T-promoter and PGL3-promoter constructs (*P <*0.001 and *P <*0.001, respectively), indicating that the mutant allele C of rs13005431 may decrease promoter activity. In addition, no significant difference was observed between the PGL3-rs13005431T-promoter group and the PGL3-promoter group (*P* =0.077), suggesting that the wild type allele T of rs13005431 had no significant effect on promoter activity.

### Expression Levels of *NFE2L2* in Neutrophils With Different Genotypes of rs13005431

**Figure 3:**
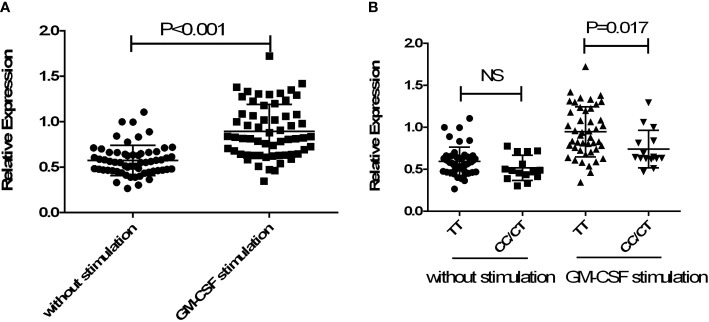
Gene expression analysis of *NFE2L2* in neutrophils. **(A)** Comparison of the mRNA expression level of *NFE2L2* between GM-CSF stimulated and without stimulated human neutrophils. **(B)** Comparison of the effects of different genotypes of rs13005431 on the mRNA expression level of *NFE2L2* in the GM-CSF stimulated group and unstimulated group. NS, No statistical difference.

To further confirm whether allele C of rs13005431 influences the promoter activity of the *NFE2L2* gene, 60 healthy subjects were enrolled. The relative expression levels of *NFE2L2* in neutrophils were analyzed according to different genotypes under basal and GM-CSF activated conditions. As shown in **Figure 3A**, the mRNA expression levels of *NFE2L2* in GM-CSF activated neutrophils were significantly increased compared with the unstimulated group (*P <*0.001). As shown in **Figure 3B**, subjects with genotype TC/CC had lower relative expression level than subjects with genotype TT in the GM-CSF activated group (*P* =0.017) while no difference was found when the neutrophils were unstimulated (*P* =0.123).

## Discussion

TB is a complex inflammatory process caused by *Mycobacterium tuberculosis* (*M. TB*). Active TB in adults is largely due to reactivation of primary infection (25), thus management of LTBI has been promoted for achieving the goals of the End-TB strategy (26). Several gene polymorphisms have shown significant associations with TB and LTBI susceptibility (27), which may help to reveal new aspects of the pathogenesis of TB and discover potential diagnostic genetic markers. It has been suggested that oxidant/antioxidant imbalance is related to TB (7, 8). Nrf2, a critical regulator of antioxidant defenses, has been shown to play a protective role in many lung diseases such as COPD, bleomycin-induced pulmonary fibrosis and so on (28, 29). Palanisamy et al. have shown that deficiency of Nrf2 was associated with progressive oxidative stress in guinea pigs infected with TB and antioxidant drugs could be a beneficial adjunct to anti-TB treatment (14). One study suggested that an Nrf2-mediated 17-gene signature can be used to distinguish TB patients from healthy controls, LTBI, pneumonia, or lung cancer, as well be used as an indicator of the anti-TB response (30). Here, we performed the first study of the relationship of *NFE2L2* SNPs with TB and LTBI susceptibility in the Chinese Han population.

Our results showed that tagSNPs of *NFE2L2* affect the susceptibility to TB and LTBI. The rs13005431_C and rs2364723_G alleles of *NFE2L2* were both risk factors for TB. The haplotype analysis results also showed the association between rs13005431 and TB. The replication study revealed that rs6726395_A was associated with increased risk of active TB when comparing with the LTBI group, while none of the SNPs were associated with TB infection. Different genetic associations between TB and LTBI have been observed for SNPs of genes such as *IRGM* (27), *FOXO3* (31), and *TLR9 *(32). These data indicate that the underlying genetic mechanisms might differ between susceptibility to LTBI and active TB. It is interesting that *NFE2L2* SNPs are also associated with diabetes mellitus (33, 34), which is one of most common comorbidities of TB. Therefore, *NFE2L2* dysfunction might serve as the common mechanism leading to TB and other diseases such as diabetes mellitus.

Additionally, we performed subgroup analysis according to TB location, gender and smoking status. Our results revealed that rs13005431_C was associated with increased risk of TB in the EPTB subgroup rather than in PTB subgroup. It has been reported that genetic susceptibility to TB differs between PTB and EPTB (31). Moreover, different mechanisms of immunity in different locations were found in mouse models (35). Our stratified results strengthen the hypothesis that polymorphisms involved in immunological mechanisms and the pathology of different TB types are different. Complicating factors such as gender, smoking and years after primary infection might influence the reactivation site (36). In another subgroup analysis by gender, rs13005431_C, rs2364723_G and rs6726395_A were found to be significant risk alleles in females but not in males. Multiplicative and additive interactions between female sex and rs13005431 genotypes were also observed. Previous studies have revealed that there were gender-specific associations between *NFE2L2* gene variants and several diseases (37, 38). Socio-behavioral, cultural factors and gonadal steroids may relate to the gender differences in TB (39, 40).

In subgroup analysis by smoking status, the rs13005431_C and rs2364723_G alleles showed relationships with increased TB risk in the non-smoking subgroup. It is well known that smoking confers a higher risk of *M. TB tuberculosis* infection, TB development and progression (41–43). The MDR results also revealed smoking-rs13005431 interactions influence TB susceptibility, with smoking contributing to high risk of TB. Animal models and human studies have shown that Nrf2 and downstream activated genes play a critical role in defending against the oxidative stress of cigarette smoke (44, 45). Nrf2 was activated and numerous antioxidant enzymes were increased in healthy smokers (44, 46, 47). The relatively lower antioxidant responses of non-smoking individuals may account for susceptibility to TB in the non-smoking subgroup.

Introns have been shown to influence gene transcription in some reports (23, 48). Intronic polymorphisms may regulate the transcriptional activity or mRNA splicing after transcription. Based on our association results, rs13005431 was investigated using dual-luciferase reporter assays to determine the influence of the SNP on transcriptional activity. The normalized luciferase activity of plasmid PGL3-rs13005431C-promoter was lower than that of PGL3-rs13005431T-promoter, suggesting that the C allele of rs13005431 decreases promoter activity. Neutrophils are one of the main phagocytes involved in *M. TB* infection. In the sputum and bronchoalveolar lavage fluid of active TB patients, neutrophils are the most prevalent cell type. Therefore, neutrophils are representative of immune response cells associated with *M. TB* infection. A previous study showed that in a chronic infectious granulomatous disease similar to the pathology of TB, the expression and nuclear translocation of *NFE2L2* in neutrophils increased in early granulomatous lesions, but this phenomenon was not found in macrophages (49). Therefore, Nrf2 may be involved in protecting neutrophils from oxidative stress and controlling inflammation to a certain extent (49). Since neutrophils play an important role in the oxidative stress of the microbicidal response to TB, we speculate that the role of Nrf2 in *M. TB* infection may be mainly reflected in protecting neutrophils from oxidative stress in TB granuloma (50). Therefore, we compared the mRNA level of *NFE2L2* in neutrophils with different genotypes to confirm the effect of rs13005431 on *NFE2L2* transcriptional activity. After stimulation by GM-CSF, neutrophils with the TC/CC genotype of rs13005431 expressed reduced *NFE2L2* mRNA level compared with neutrophils with the TT genotype. From all of the above results, we can speculate that the C allele of rs13005431 may increase the risk of TB by lowering the promoter activity of *NFE2L2* and reducing its mRNA level.

The potential weaknesses of our study should be listed. Firstly, we did not further investigate the specific transcription factors binding to the sequence located around rs13005431 and thus clarify the underlying mechanism for the increased TB susceptibility. Secondly, we did not directly provide evidence that during *M.TB* infection, rs13005431 would be an important player or even an expression quantitative trait locus (eQTL). Finally, since our research was limited to the Chinese Han population, follow up studies in different populations are needed to validate our results.

## Conclusion

In conclusion, we have observed significant associations between *NFE2L2* variants and TB susceptibility. Further experiments suggested the potential mechanism: allele C of rs13005431 decreased the transcriptional level of *NFE2L2*. This study may thus pave the way for new treatment modalities targeting anti-oxidative mechanisms in TB.

## Data Availability Statement

The raw data supporting the conclusions of this article will be made available by the authors, without undue reservation, to any qualified researcher. All data excel files are available from the Figshare database (https://figshare.com/s/917cdb79772f89aee2f9).

## Ethics Statement

The studies involving human participants were reviewed and approved by the ethics committee of the West China Hospital of Sichuan University in China. The patients/participants provided their written informed consent to participate in this study.

## Author Contributions

Conceived and designed the study: GJ and J-QH. Analyzed the data: QL, MZ, YW, SW, GC, and J-QH. Wrote the manuscript: GJ, MZ, QL, AS, and J-QH. All authors contributed to the article and approved the submitted version.

## Funding

This work was supported by the Research Fund from the National Scientific and Technological Major Project of China (Beijing, China) Grant 2012ZX10004-901, the National Natural Science Foundation of China (Beijing, China) Grants 81072432, 81170042, and 81370121, Science & Technology Department of Sichuan Province (Chengdu, Sichuan, China) Grant 2012SZ0126, Post-Doctor Research Project, West China Hospital, Sichuan University (2019HXBH085), and Investigator-Initiated Clinicaltrial, West China Hospital, Sichuan University (HXCR20001).

## Conflict of Interest

The authors declare that the research was conducted in the absence of any commercial or financial relationships that could be construed as a potential conflict of interest.

## Abbreviations

M.TB, *Mycobacterium tuberculosis*; TB, tuberculosis; LTBI, latent tuberculosis infection; TST, tuberculin skin test; IGRAs, Interferon-gamma release assays; BCG, bacille Calmette–Guérin; Nrf2, Nuclear factor-erythroid 2 (NF-E2)-related factor 2; HC, healthy controls; PTB, pulmonary TB patients; EPTB, extra-pulmonary patients; PCR, Polymerase chain reaction; OR, Odds Ratios; 95%CI, 95% confidence intervals; LD, Linkage disequilibrium; HWE, Hardy-Weinberg equilibrium; MDR, multifactor dimensionality reduction; RERI, relative excess risk; AP, attributable proportion; S, synergy index; eQTL, Expression quantitative trait loci.
