# Supplementary material for: Functional Polymorphism in the NFE2L2 Gene Associated With Tuberculosis Susceptibility
Source: Front Immunol. 2021 May 24;12:660384. doi: 10.3389/fimmu.2021.660384 (PMC8181729; doi:10.3389/fimmu.2021.660384)

***Supplementary Material***

**Functional polymorphism in the *NFE2L2* gene associated with tuberculosis susceptibility**

Guiyi Ji^1,2,+^, Miaomiao Zhang^1,+^, Qianqian Liu^3,+^, Shouquan Wu^1^, Yu Wang^1^, Guo Chen^3^, Andrew J Sandford^4^, Jian-Qing He^1*^

*Corresponding author

Dr. Jian-Qing He.

1. mail: [jianqhe@gmail.com](mailto:jianqhe@gmail.com), jianqing_he@scu.edu.cn.

Supplementary Table 1: List of genotyping related primers

| SNPs | Primer sequences (5’-3’) |
| --- | --- |
| rs10497511 |  |
| Forward | ACGTTGGATGAGGAATTGTATGGAGGTGGG |
| Reverse | ACGTTGGATGGCTGGACTTCTCTTTGGAGC |
| Single base extension primer | CTGTTCCACGGATCG |
| rs2364723 |  |
| Forward | ACGTTGGATGTTAACCCAGGCTTGAGGAAC |
| Reverse | ACGTTGGATGTTCCTCTGTCCTGACTGAAG |
| Single base extension primer | GTTTTGTGTCAATATTTCCTC |
| rs13005431 |  |
| Forward | ACGTTGGATGTGAGGACAATATTGACTGTG |
| Reverse | ACGTTGGATGGCAGACAAGAGATTGTCAGG |
| Single base extension primer | GGTCTGAAATAAAGCAAAATTCTATA |
| rs6726395 |  |
| Forward | ACGTTGGATGGATAATATTTGAGGGGTTTGG |
| Reverse | ACGTTGGATGTCACGTGCCTCATTTGATCC |
| Single base extension primer | TGATCCACTAACATCTGTA |
| rs1962142 |  |
| Forward | ACGTTGGATGTTCAGAGGACTTTCCTTCTC |
| Reverse | ACGTTGGATGAGGCACAGTCTTACAGAACC |
| Single base extension primer | ACCACACAATATTCAGAGCTTG |

Supplementary Table 2: List of primer pairs

| Primer name | Primer sequences (5’-3’) | Length of product |
| --- | --- | --- |
| Primers for RT-PCR |  |  |
| NFE2L2-Forward | TTCTGTTGCTCAGGTAGCCCC |  |
| NFE2L2-Reverse | TCAGTTTGGCTTCTGGACTTGG | 161bp |
| B2M- Forward | GCCGTGTGAACCATGTGACTTT |  |
| B2M- Reverse | TGCGGCATCTTCAAACCTCCAT | 92bp |
| Primers for PGL3-rs13005431T-prom |  |  |
| rs13005431T-Forward | CTAGCTAGCCTTAGTCAGCCCTCAAACACC |  |
| rs13005431T-Reverse | CCGCTCGAGGCTCGCAAGGCCCAATT | 651 bp |
| Primers for PGL3-rs13005431C-prom |  |  |
| rs13005431C-Forward | GCAAAATTCTATACGCCTTAACAGTGGG |  |
| rs13005431C-Reverse | GCCCACTGTTAAGGCGTATAGAATTTTG | 5661 bp |
| B2M, beta-2-microglobulin | | |

Supplementary Table 3 Associations between haplotypes and tuberculosis susceptibility in the discovery study.

| Cohort | Haplotype | TB(frequency) | Control(frequency) | Chi^2^ | *P* | OR (95%CI) |
| --- | --- | --- | --- | --- | --- | --- |
| Discovery study | rs10497511-rs13005431 |  |  |  |  |  |
|  | A C | 204.5(0.162) | 153.8(0.127) | 6.16 | **0.013** | 1.33 (1.06-1.67) |
|  | A T | 751.5(0.595) | 775.2(0.640) | 5.04 | **0.025** | 0.83 (0.71-0.98) |
|  | G T | 305.45(0.242) | 282.8(0.233) | 0.26 | 0.609 | 1.05(0.87-1.26) |
|  | Global result | 1264 | 1212 | 7.40 | **0.025** |  |
|  | rs1962142-rs6726395 |  |  |  |  |  |
|  | A A | 275(0.216) | 254.4(0.209) | 0.15 | 0.700 | 1.04 (0.86-1.26) |
|  | G A | 255(0.200) | 218.6(0.180) | 1.63 | 0.201 | 1.14(0.93-1.39) |
|  | G G | 737(0.579) | 734.4(0.604) | 1.81 | 0.179 | 0.90(0.76-1.05) |
|  | Global result | 1272 | 1216 | 2.17 | 0.338 |  |

Abbreviations: OR, odds ratio; CI, confidence interval.

Supplementary Table 4 Gene-gene and gene-environment interactions between tagSNPs of *NFE2L2* and tuberculosis susceptibility using MDR analysis.

| Best model | Bal.Acc.CV Testing | CV Consistency | p value* |
| --- | --- | --- | --- |
| smoking | 0.5168 | 9/10 | 0.229-0.230 |
| smoking, rs13005431 | 0.5569 | 10/10 | **0.001-0.002** |
| smoking, sex, rs13005431 | 0.5503 | 8/10 | **0.023*** |
| smoking, sex, rs13005431, rs2364723 | 0.5209 | 3/10 | 0.4610 |
| smoking, sex, rs13005431, rs2364723, rs6726395 | 0.5451 | 8/10 | 0.0820 |
| smoking, sex, rs13005431, rs1962142, rs2364723, rs6726395 | 0.5558 | 10/10 | **0.023-0.024** |
| smoking, sex, rs10497511, rs13005431, rs1962142, rs2364723, rs6726395 | 0.5600 | 10/10 | **0.013-0.014** |

*The p value of each best model was calculated after 1000-fold permutation testing, applying Testing accuracy and CV Consistency in the MDR permutation test module.

Supplementary Table 5 Interaction of significant SNP-by-sex in the discovery study under dominant and allelic models.

| variants×SNP | Multiplicative interaction analysis | | Additive interaction analysis | | |
| --- | --- | --- | --- | --- | --- |
|  | p value | OR 95%CI | RERI 95%CI | AP 95%CI | S 95%CI |
| female×rs13005431 TC+CC | **0.006** | 2.047(1.227-3.415) | **2.845(0.247-5.443)** | **0.396(0.160-1.155)** | **1.850(1.155-2.963)** |
| female×rs13005431 C | **0.006** | 1.899(1.203-2.998) | **3.524(0.746-6.303)** | **0.425(0.218-0.631)** | **1.934(1.270-2.944)** |
| female×rs2364723 CG+GG | **0.031** | 1.770(1.055-2.969) | -0.058(-1.185-1.068) | -0.016(-0.323-0.291) | 0.979(0.647-1.480) |
| female×rs2364723 G | **0.032** | 1.414(1.030-1.942) | -0.260(-1.233-0.713) | -0.062(-0.298-0.175) | 0.925(0.693-1.24) |
| female×rs6726395 AG+AA | **0.001** | 2.179(1.362-3.486) | -0.137(-0.869-1.144) | -0.041(-0.256-0.338) | 1.062(0.679-1.661) |
| female×rs6726395 A | **0.002** | 1.664(1.204-2.299) | -0.075(-0.889-1.039) | -0.018(-0.212-0.248) | 1.024(0.751-1.397) |

Abbreviations: OR, odds ratio; CI, confidence interval; RERI, relative excess risk due to interaction; AP, attributable proportion due to interaction; S, synergy index. For additive interaction analysis, if there is no additive interaction, RERI and AP are equal to 0 and S is equal to 1.

| Supplementary Table 6. The results of bioinformatics prediction. | | | | | | | | | |
| --- | --- | --- | --- | --- | --- | --- | --- | --- | --- |
| SNPs | Location | Transcriptional regulation | | | | | |  |  |
|  |  | Non-risk allele binding | Risk allele binding | Non-risk allele binding | Risk allele binding | Prediction | Evolutionary conservatism | Protein coding | Splicing regulation |
|  |  | (TRANSFAC) | (TRANSFAC) | (JASPAR) | (JASPAR) | (GLODEN path) |  |  |  |
| rs13005431 | 第一内含子 | - | Pax-4 | FOXL1 | FOXC1 | exist | conserved | - | - |
| rs13001694 | 第一内含子 | - | - | - | - | exist | conserved | - | - |
| rs2364723 | 第一内含子 | - | - | - | - | - | - | - | - |
| rs2364722 | 第一内含子 | - | - | - | - | exist | conserved | - | - |
| rs2886161 | 第一内含子 | Oct-1 | - | - | - | exist | - | - | - |
| rs6726395 | 第一内含子 | - | - | - | - | exist | - | - | - |

Figure Legends

Supplementary Figure 1 Schematics of the three plasmids used in the luciferase experiments: PGL3-prom, PGL3-rs13005431T-prom, and PGL3-rs13005431C-prom.


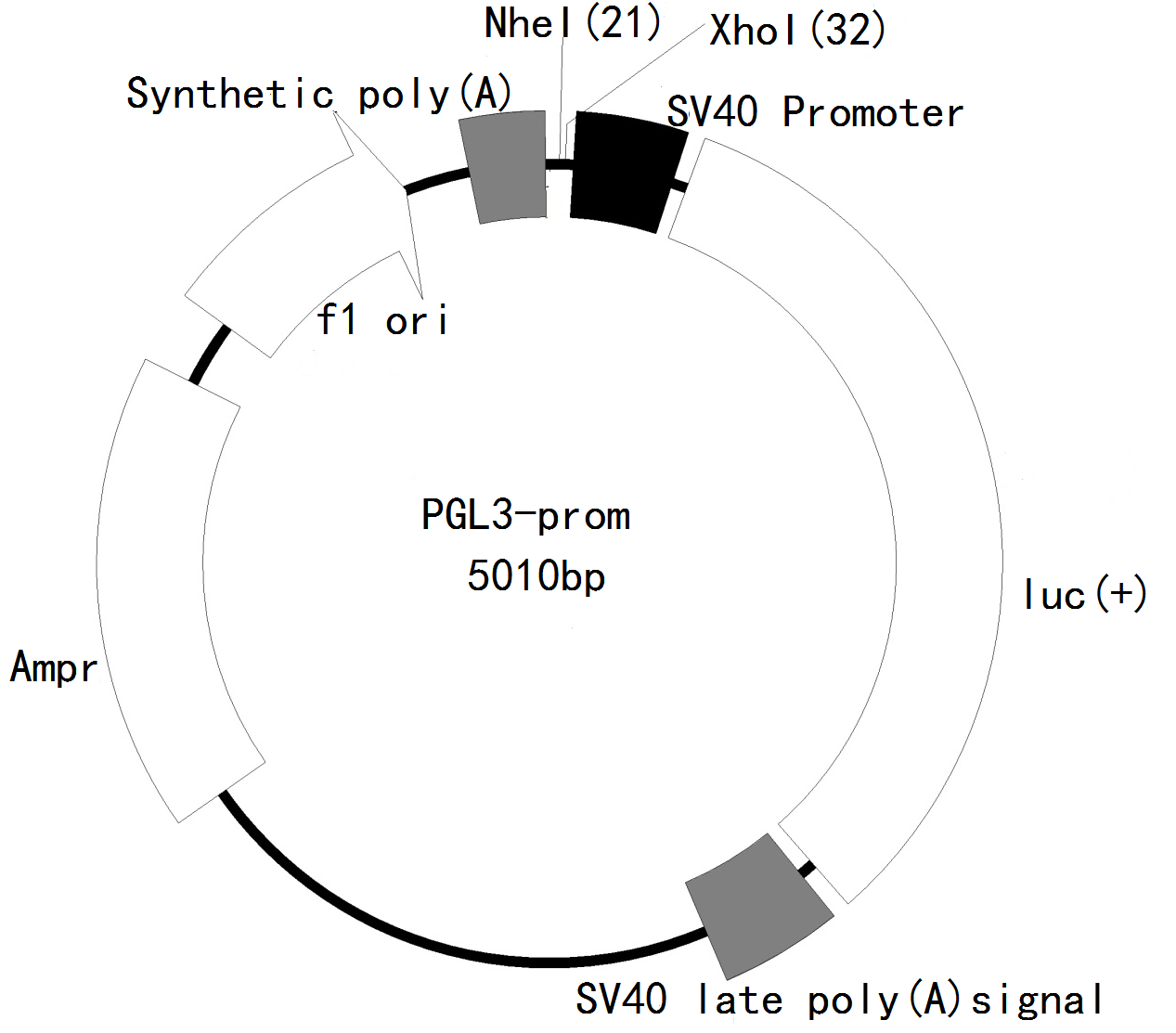


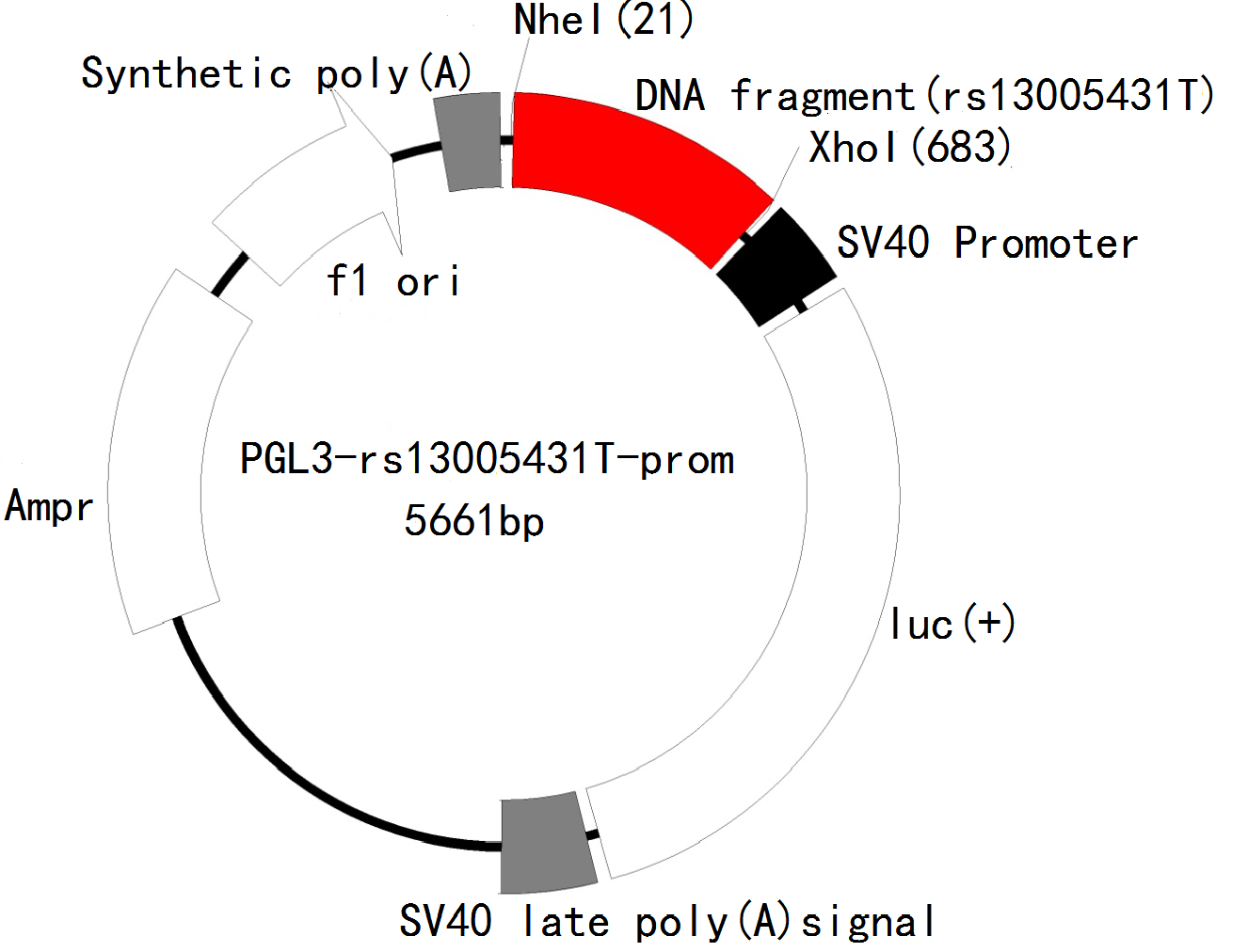


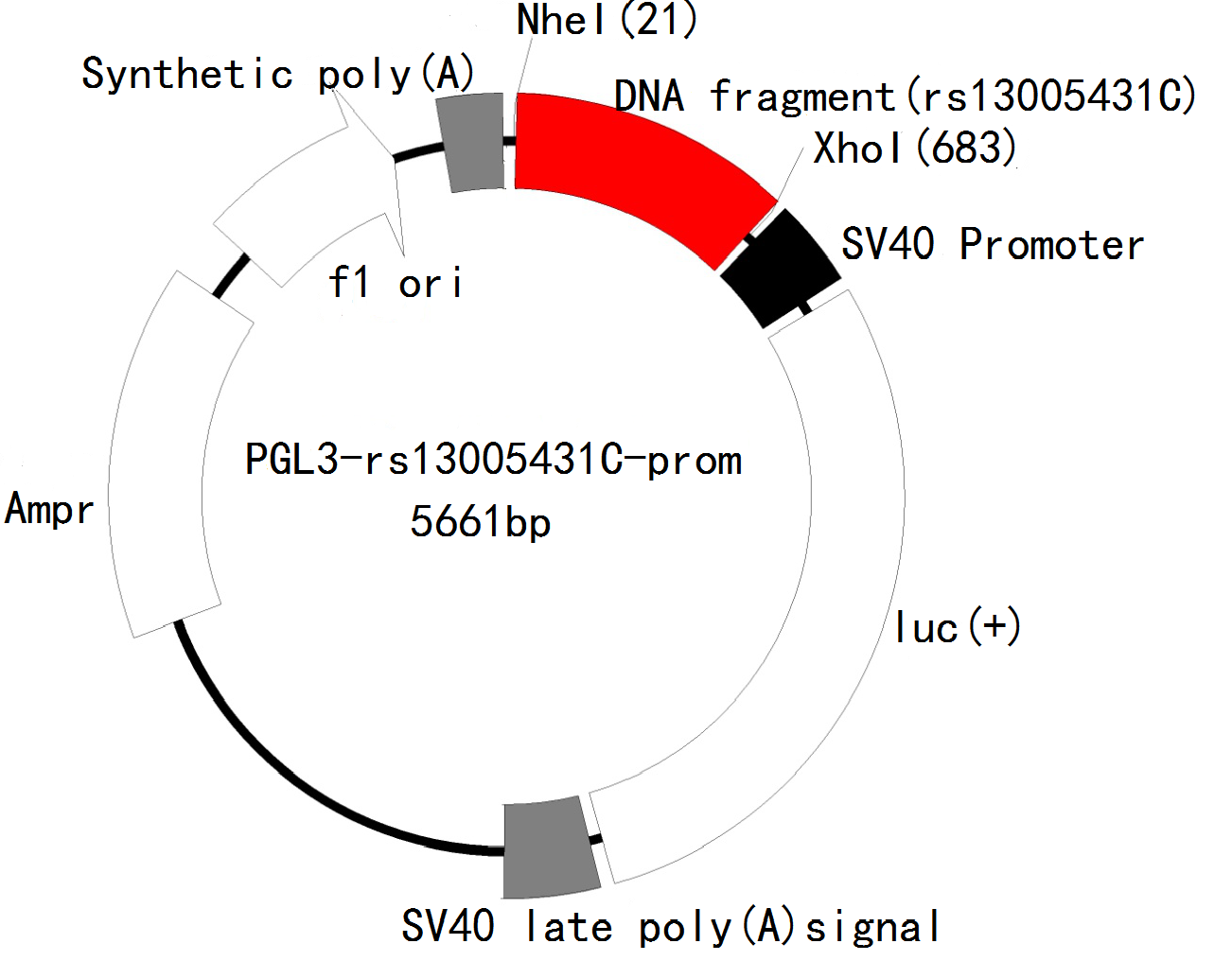


Supplementary Figure 2 Linkage disequilibrium (LD) of all five tagSNPs displayed in the form of r^2^. Each colored cell is related to the strength of LD between the corresponding two markers. The number in each cell represents the LD parameter r^2^ (×100).


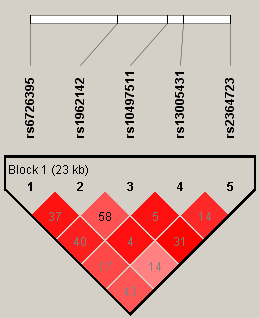


Supplementary Figure 3 Distribution of high-risk and low-risk models associated with TB after MDR analysis. The numbers of TB patients (left bars) and HC subjects (right bars) are shown in cells. Dark gray cell indicates high-risk models (more TB patients than controls) and the light gray cell indicates low-risk models (less TB patients than controls).


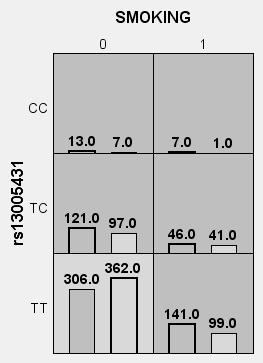

Supplement: Supplementary file 1 [file DataSheet_1.docx]
